# *SHISA3*, an antagonist of the Wnt/β-catenin signaling, is epigenetically silenced and its ectopic expression suppresses growth in breast cancer

**DOI:** 10.1371/journal.pone.0236192

**Published:** 2020-07-21

**Authors:** Naveed Shahzad, Tehreem Munir, Mariam Javed, Fareeda Tasneem, Bilal Aslam, Moazzam Ali, Zeeshan Mutahir, Muhammad Akhtar Ali, Muhammad Umer, Munir Ahmad, Kokab Farooq, Umair Hassan, Tanveer Mustafa, Rana Salman Anjum, Abdul Rauf Shakoori

**Affiliations:** 1 School of Biological Sciences, University of the Punjab, Lahore, Pakistan; 2 Department of Zoology, University of the Punjab, Lahore, Pakistan; 3 Department of Microbiology, Government College University, Faisalabad, Pakistan; 4 Institute of Biochemistry and Biotechnology, University of the Punjab, Lahore, Pakistan; 5 Queensland Micro- and Nanotechnology Centre, Griffith University, Nathan, Australia; 6 Department of Histopathology, Fatima Jinnah Medical University, Lahore, Pakistan; 7 School of Life Sciences, Forman Christian College University, Lahore, Pakistan; Chinese University of Hong Kong, HONG KONG

## Abstract

Breast cancer (BC) is the foremost cause of cancer related deaths in women globally. Currently there is a scarcity of reliable biomarkers for its early stage diagnosis and theranostics monitoring. Altered DNA methylation patterns leading to the silencing of tumor suppressor genes are considered as an important mechanism underlying tumor development and progression in various cancer types, including BC. Very recently, epigenetic silencing of *SHISA3*, an antagonist of β-catenin, has been reported in various types of tumor. However, the role of *SHISA3* in BC has not been investigated yet. Therefore, we aimed at evaluating the contribution of *SHISA3* in BC causation by analyzing its expression and methylation levels in BC cell lines (MDA-MB231, MCF-7 and BT-474) and in 103 paired BC tissue samples. The *SHISA3* expression and methylation status was determined by qPCR and methylation specific PCR (MSP) respectively. The role of *SHISA3* in BC tumorigenesis was evaluated by proliferation and migration assays after ectopic expression of *SHISA3*. The association between *SHISA3* hypermethylation and clinicopathological parameters of BC patients was also studied. The downregulation of *SHISA3* expression was found in three BC cell lines used and in all BC tissue samples. However, *SHISA3* promoter region was hypermethylated in 61% (63/103) tumorous tissues in comparison to the 18% of their matched normal tissues. The 5-aza-2’-deoxycytidine treatment restored *SHISA3* expression by reversing promoter hypermethylation in both MDA-MB231 and MCF-7 cells. Furthermore, ectopic expression of *SHISA3* significantly reduced the proliferation and migration ability of these cells. Taken together, our findings for the first time reveal epigenetic silencing and tumor suppressing role of *SHISA3* in BC. Henceforth, this study has identified *SHISA3* as potentially powerful target for the development of new therapies against BC, as well as novel diagnostic and therapy response monitoring approaches.

## 1. Introduction

Breast cancer (BC) is one of the most widespread cancer with continuously increasing incidence rate and the foremost reason of deaths among women across the world [1]. According to WHO, more than 627,000 women died of BC around the world in 2018 only, accounting for around 15% of all tumor related mortalities among women. One of the major reasons for high BC related deaths is late diagnosis especially in resource limited settings where majority of women are diagnosed during late stages due to weak and costly health care systems. While these data indicate the inefficiency of current strategies, they also highlight the urgency to the development of more reliable approaches for early stage tumor diagnosis and therapy response monitoring for BC. Furthermore, it is desirable that the future diagnostic platforms should suit the needs of point-of-care (POC) and resource limited health care settings. In comparison with current gold standard BC diagnosis methods, like mammography, molecular biomarkers-based approaches are relatively easier to be integrated into portable and easy to use devices for POC applications [2, 3].

The initiation and progression of BC is marked by certain genetic and epigenetic alterations, which can deregulate the cancer related genes, for instance, activation of oncogenes and suppression of tumor suppressor genes (TSGs) [4, 5]. While these changes can be exploited for development of new diagnostic and therapeutic approaches, cancers being highly heterogeneous, there is an ongoing need for identification of novel cancer specific molecular aberrations. Promoter hypermethylation is the leading epigenetic alteration linked to the transcriptional inactivation of several TSGs and is considered as an early event in carcinogenesis [6]. Several biomarkers based on the peculiar DNA methylation patterns have also been described for diagnosis and prognosis of multiple cancer types including BC [7]. Furthermore, reversibility of methylation phenomenon makes it an attractive target for cancer cure [8]. Therefore, identification of novel BC related DNA methylation biomarkers would be of great significance in BC diagnostics and therapeutics.

The *SHISAs* family of endoplasmic reticulum residing proteins, comprises of eight members (*SHISA2-SHISA9*) in vertebrates and are mainly involved in head formation of non-human species including Xenopus, mouse, and chicken [9, 10]. These proteins are reported to modify the *Wnt* and FGF signaling by hindering the maturation and transportation of their receptors to the cell surface [11, 12]. *Wnt* signaling pathway is believed to play a predominant role in the development of mammary gland at both antenatal and postnatal stages. *Wnt*/β-Catenin pathway as well as various *Wnt* ligands are known to participate in the initiation and formation of mammary rudiments at antenatal stage. Whereas, *Wnt* proteins have been shown to mediate ductal growth in mammary tissues in an ovarian hormones independent manner [13, 14]. Likewise, unusual activation of *Wnt* pathway has been associated with the development of BC, especially triple negative BC [15]. Stabilization of β-Catenin, which is a central regulator of *Wnt* pathway, is frequently observed in BC. Similarly, a range of studies have shown that various *Wnt* ligands, receptors, and transducers are overexpressed while *Wnt* antagonists are under-expressed frequently in BC [16, 15]. However, despite their role as antagonists of *Wnt* signaling, very few members of *SHISA* proteins have been investigated in humans for their association with diseases particularly cancer.

*SHISA3* was probably among the first few members of *SHISA* family identified to play role in human carcinogenesis. Chen et al. [17] reported that *SHISA3* helps in suppression of tumorigenesis, invasion, and metastasis by promoting the degradation of β-catenin [17]. Later on, *SHISA3* silencing was also observed in colorectal cancer as a consequence of promoter hypermethylation [18]. Subsequently, epigenetic inactivation of *SHISA3* was also described in laryngeal squamous cell carcinoma [7] and nasopharyngeal carcinoma [19]. In all of these studies, tumor suppressor nature of *SHISA*3 and its hypermethylation was found to be crucial in the diagnosis and prediction of the clinical outcome of various cancer types. Nevertheless, the tumor suppressing action and promoter methylation of *SHISA3* has not been fully investigated yet in other types of cancer including BC.

Previous research has shown that many cancers share common molecular mechanisms [20, 21]. Identification of such overlapping molecular mechanisms that may be dysregulated in multiple cancers may help in developing diagnostic platforms and therapeutic targets with broad applications. Since *SHISA3* expression is dysregulated in multiple cancers, the present study aimed at investigating the status of its expression in BC as well as its potential role in BC pathogenesis. Towards this end, we analyzed the expression of *SHISA3* gene in BC cell lines and primary tissues. The mechanism of *SHISA3* silencing and its clinical relevance in BC was also evaluated in this study. Herein we report for the first time that promoter hypermethylation mediated downregulation of *SHISA3* gene is a frequent event in BC. Our findings may pave the way for development of novel diagnostic and theranostics approaches for management of BC.

## 2. Materials and methods

### 2.1 Breast cancer cell lines and patient tissue samples

Breast cancer cell lines representing three different molecular subtypes; MCF-7 (Luminal A, ER^+^, PR^+/-^, HER2^-^), BT-474 (Luminal B, ER^+^, PR^+/-^, HER2^+^), MDA-MB231 (Claudin-low, ER^-^, PR^-^, HER2^-^) and non-tumorigenic mammary epithelial cells from normal breast tissue, were used in this study. The MCF-7 and MDA-MB231 cell lines were purchased from ATTC, whereas, BT-474 was obtained from cell lines bio bank of Panjwani Center for Molecular Medicine and Drug Research (PCMD), Karachi, Pakistan. The complete DMEM medium (Dulbecco’s Modified Eagle Medium (DMEM) (Gibco) supplemented with 10% Fetal Bovine Serum (FBS) (Gibco) and 1% penicillin/streptomycin (Gibco) was used to grow all cell lines and the cells were incubated at 37°C, 5% CO₂ and 70% humidity.

In the present study, 103 sporadic BC patients who never received anti-tumor therapy and underwent surgery for primary tumor resection at Ganga Ram & Mayo Hospital Lahore, Pakistan from January 2016 to December 2018 were recruited. In total, 103 primary BC and adjacent normal mammary tissues were collected directly after surgical excisions from these patients. The tissue samples were either directly frozen in liquid nitrogen or stored in RNA later™ (ThermoFisher Scientific, USA) until DNA or RNA extraction. Furthermore, hematoxylin and eosin stained sections of all tissue samples were subjected to histological diagnosis by a specialist pathologist. Histopathological characteristics of representative human breast (normal and cancerous) tissues are described in **S1 Fig** Clinicopathological data for instance age, histological grade, clinical stage and tumor size etc. were also collected for all samples. The present study was performed after authorization of Institutional Ethical Review Committee (IERC) of School of Biological Sciences, University of the Punjab, Lahore, Pakistan. Patients recruited for the study were provided informed consent material in both English and Urdu (native) languages. All the participating patients provided written informed consent.

### 2.2 RNA extraction and *SHISA3* mRNA quantification

Total RNA was extracted from BC cell lines and 19 tissue samples by using PureLink™ RNA mini Kit (Invitrogen, USA). RevertAid First Strand cDNA Synthesis Kit (Thermo Scientific, USA) was used to convert extracted RNA into cDNA, according to the manufacturer’s protocol. For expression analysis of *SHISA3*, Flourogenic Quantitative Real Time PCR (qPCR) was performed by using SYBR green master mix (Thermo Fisher Scientific) in PikoReal real time detection system (Thermo Fisher Scientific). The cycling conditions for qPCR assay were as follows: 95°C for 10 min; 40 (*SHISA3*) or 35 (*GAPDH*) cycles of 95°C for 30s, 57°C (*SHISA3*) and 59°C (*GAPDH*) for 30s and 72°C for 30s and final extension for 5 min at 72°C. After normalization with internal control (*GAPDH1*), the differences in *SHISA3* transcripts level were measured by adopting (2^-ΔΔCT) method described elsewhere [22] and expressed as relative fold change. All samples were assayed in triplicate in three independent experiments. The *SHISA3* and *GAPDH* specific primer sets are enlisted in **Table 1**.

**Table 1:** List of all primers used in the study.

Primers	Sequence (5’-3’)	Amplicon size (bps)
qPCR primers		
*SHISA3*		
Forward	CTGCGCAGCCTGTCTACGTC	148
Reverse	CTGCGGAGTGAGAAGCGGAT	
*GAPDH*		
Forward	AAGGTGGTGAAGCAGGCGT	130
Reverse	GAGGAGTGGGTGTCGCTGTT	
MSP primers		
*SHISA3* Methylated		
Forward	AGAGGTGATCGGTAATTTTTTAGTC	204
Reverse	CCTATTACACAAACTCAAACTCGTT	
*SHISA3* Un-Methylated		
Forward	GAGGTGATTGGTAATTTTTTAGTTG	203
Reverse	CCTATTACACAAACTCAAACTCATT	
Cloning primers		
*SHISA3*		
Forward	TTTTTAAAGCTTATGAGGGCTCTGCTGGC	700
Reverse	ATTAAAGCGGCCGCTCAACTGGAACTGAAGTCTGGACA	

### 2.3 Genomic DNA isolation and bisulfite conversion

Genomic DNA was extracted from BC cell lines and tissue samples using PureLink™ Genomic DNAmini Kit (Invitrogen Inc.) following manufacturer’s protocol. After that, 2 μg of DNA from each sample was subjected to bisulfite conversion by using EpiJET Bisulfite Conversion Kit (ThermoFisher Scientific, USA) as per manufacturer’s instructions. The bisulfite-treated DNA was kept at -80°C till further use.

### 2.4 Methylation-Specific Polymerase chain reaction (MSP) assay

In order to investigate the methylation status of selected CpG site of *SHISA3* promoter region, MSP assay was performed on BC cell lines and 103 BC and adjacent normal tissue samples. The methylation and un-methylation specific primer pairs used in MSP assay are described in **Table 1**. To verify primer specificity, we performed a standard PCR where methylation and un-methylation specific primers were used to amplify the commercially available methylated and un-methylated control templates (Zymo Research^TM^,) and vice versa. For both methylated and un-methylated primers water was used as negative control. Each 25μl MSP reaction contained 200 ng (2μl) of bisulfite converted DNA template, 1X reaction buffer, 25mM MgCl_2_, 2.5mM dNTPs, 1𝜇M each of forward and reverse primers and 5U of Hot start Taq polymerase (ThermoFisher Scientific). The PCR cycling conditions were; initial denaturation at 94°C for 10 min followed by 40 cycles of 94°C for 30 seconds, 56°C for 30s, 72°C for 30s and final extension at 72°C for 5 min. Commercially available methylated and un-methylated DNA templates were used as a positive control for methylation and un-methylation specific primers respectively and water blank reaction as a negative control was used for each type of reactions. The PCR products were examined on 2% agarose gel stained with ethidium bromide by visualizing under UV light. A visible DNA band from the respective reaction indicated methylation status of target CpG site.

### 2.5 *In silico* analysis of *SHISA3* expression and methylation

*SHISA3* gene expression and methylation level in breast tissues was also analyzed by *in silico* approach. Using datasets from TCGA-BRCA retrieved from online available Genomics Data Commons (GDC) database, *SHISA3* expression and methylation levels in various breast cancer types like Invasive Basal Carcinoma (IBC), Invasive Lobular Carcinoma (ILBC) and Invasive Ductal Carcinoma (IDBC) were analyzed and compared with those of normal breast tissues (NBr). RNA-Seq data was analyzed for *SHISA3* gene expression quantification at cutoff value of 1.5-fold change. Whereas, methylation (beta values) for *SHISA3* was analyzed (P<0.01) by using data generated from “Ilumina Human Methylation 27” and “Ilumina Human Methylation 450” platforms. A schematic flow chart indicating the pattern followed for online expression and methylation analysis of *SHISA3* is described in **S2 Fig**.

### 2.6 *In vitro* demethylation assay

The MDA-MB231 and MCF-7 cells at a density of 10^6^ cells/well were cultured in 6-well plate for 24 h before treatment. Both cell lines were treated with 10 and 20μM of 5’-aza-2’-deoxycytidine (Aza, Abcam) for four days with culture media replacement after every 24 h. The *SHISA3* gene expression and methylation status before and after Aza treatment were examined by qPCR and MSP respectively, as explained earlier.

### 2.7 Ectopic expression of *SHISA3* in breast cancer cells

A plasmid vector expressing full length human *SHISA3* (pcDNA3.1-*SHISA3*) was constructed by cloning *SHISA3* cDNA into the pcDNA3.1 (Invitrogen, USA) mammalian expression vector. The primers used for cloning of *SHISA3* are enlisted in **Table 1**. The cloning of *SHISA3* gene was confirmed by digesting the pcDNA3.1-*SHISA3* plasmid with Not-1 and Hind-III (Fermentas) restriction enzymes. MDA-MB231 and MCF-7 cells at 60% confluency in 6-well plates, transfected with 4μg of either empty pcDNA3.1 or pcDNA3.1-*SHISA3* vector by using Lipofectamine^®^ 2000 transfection reagent (Invitrogen). Cells were collected at several different time intervals after transfection to analyze the expression of *SHISA3* by RT-PCR.

### 2.8 Cell proliferation assay

The MDA-MB231 and MCF-7 cells were seeded in 6-well plates at 10^6^ cells per well and incubated at 37 ^0^C. For each cell line at 50 to 60% confluency, cells were transfected with either empty pcDNA3.1 or pcDNA3.1-*SHISA3* vector as described above. After 24 h, cells were gently washed with 1X PBS and transfection medium was replaced with complete DMEM medium. Three days later, after washing twice with 1X PBS, the cells were fixed with methanol, stained with 1% crystal violet for 30 min and photographed by using inverted microscope (Olympus, 1X51). The experiment was repeated thrice by using samples in duplicates each time.

### 2.9 Wound healing assay

MDA-MB231 and MCF-7 cells were seeded in 6-well plates at the concentration of 10^6^ cells per well and were grown at 37 ^0^C, 5% CO_2_ and 70% humidity. When wells were 50 to 60% confluent, cells were transfected with 4μg either empty pcDNA3.1 or pcDNA3.1-*SHISA3* vector as described above. Untransfected cells were also included as negative control. After 24 h, cells were gently washed with 1X PBS and transfected media was replaced with regular growth medium. Once the wells were confluent, a sterile 200μl pipette tip was used to gently scratch the cell monolayer across the center of the well. Detached cells were then removed by washing with the growth medium twice. The wells were replenished with fresh complete DMEM medium and photographed at 0, 24, 48, and 72 h time intervals under 4X objective lens of inverted microscope (Olympus, 1X51). The cells were counted and expressed as percentage of wound closure.

### 2.10 Statistical analyses

SPSS 16.0 software was used for all statistical analyses (SPSS Inc., Chicago, IL, USA). The data presented in this study is mean **±** SD of three independent experiments. Two tailed student’s t-test was used to compare difference in *SHISA3* expression between normal and BC tissues. Whereas, Chi square test was employed to probe the association between *SHISA3* methylation status and clinicopathological parameters of BC patients. In all analyses, P value ≤0.05 was considered as statistically significant.

## 3. Results

### 3.1 Expression of *SHISA3* in BC cell lines and primary tumors

**Fig 1:**
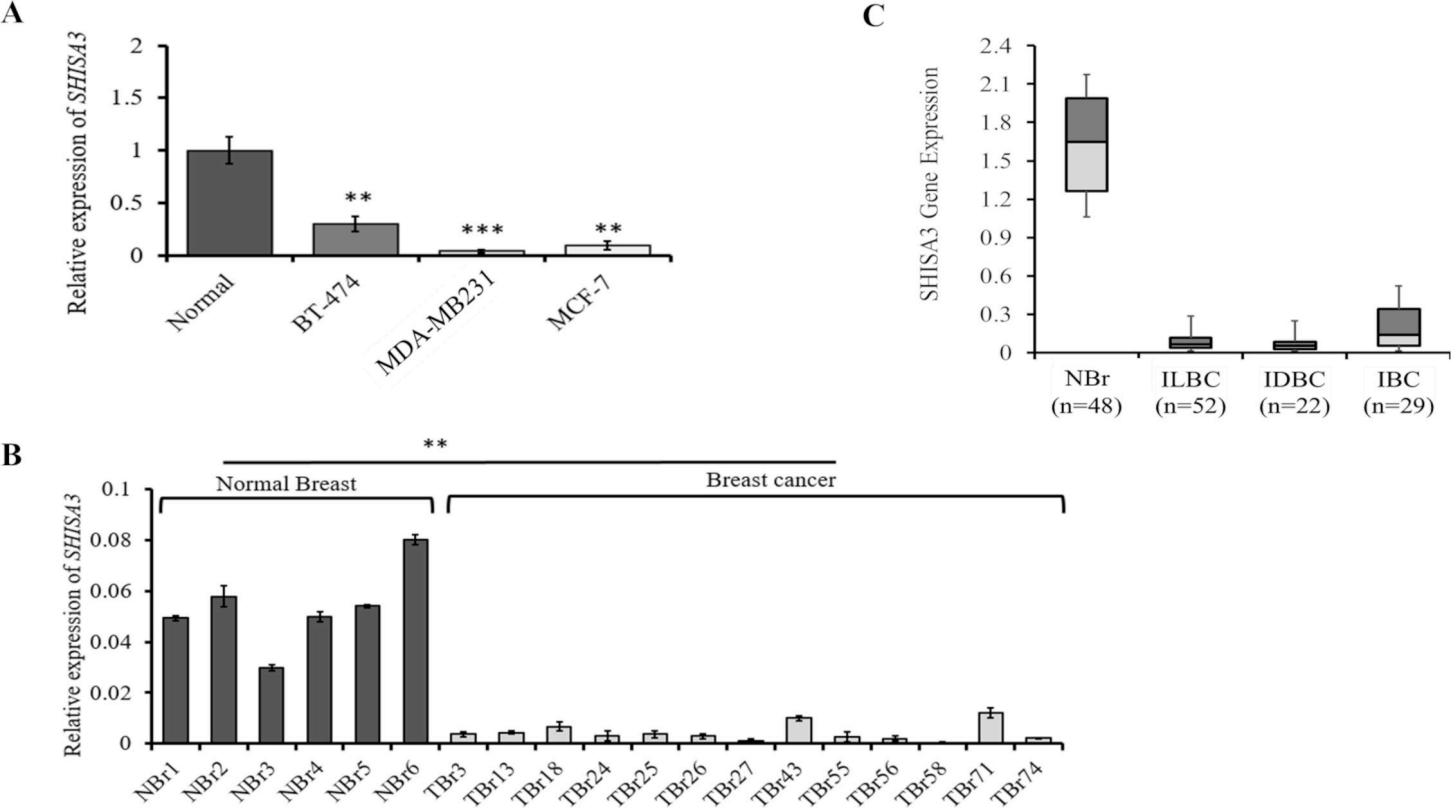
Expression analysis of *SHISA3* in breast cancer. The mRNA levels of *SHISA3* were quantified by qPCR and *in silico* analysis. A) The figure shows relative expression of the *SHISA3* in the normal cells and breast cancer cell lines. B) The bar graphs show the relative expression of the *SHISA3* genes in the normal breast (NBr) and tumor (TBr) tissues. C) *In silico* analysis of *SHISA3* expression levels in normal and various breast cancer subtypes. IBC: Invasive Basal Carcinoma, ILBC: Invasive Lobular Carcinoma, IDBC: Invasive Ductal Carcinoma and NBr: normal breast tissues.

We first determined the endogenous expression of *SHISA3* in 3 BC cell lines and cultured normal human mammary epithelial cells (Normal) by qPCR. The *SHISA3* was found to be downregulated in all three BC cell lines used as compared to the normal human mammary epithelial cells **(Fig 1A)**. The *SHISA3* expression was further evaluated in normal (n = 6) and BC (n = 19) tissues from patients. Our data revealed that the relative mRNA level of *SHISA3* was significantly lower in BC tissues than in normal breast tissues **(Fig 1B)**. GDC database contains data for almost 68 tissue types collected from 48 various projects. We carried out a comprehensive online database search at “Cancer Genome Atlas Breast Invasive Carcinoma” (TCGA-BRCA) which is now accessible through GDC website only. Data categories that we chose for current study were “DNA methylation” and “Transcriptome profiling”. In total 1095 cases of methylation array and 1097 cases of RNA-Seq analysis were available. We used methylation beta values and Fragments Per Kilo base of transcript per Million mapped reads (FPKM) values for the analysis of methylation and gene expression quantification respectively. Data for transcriptome analysis was bulk downloaded to include multiple breast cancer types from various studies and filtered for corresponding *SHISA3* probes to analyze it expression. Statistical analysis revealed that mRNA level of *SHISA3* gene was significantly lower in various subtypes of BC (20 times in ILBC, 22 times in IDBC and 8.4-fold in IBC) as compared to normal breast tissues **(Fig 1C)**.

### 3.2 Methylation status of *SHISA3* in BC and paired normal tissues

For MSP analysis, we first evaluated the efficiency and specificity of the *SHISA3* methylation specific and un-methylation specific primer sets. Our results revealed that both the primer sets used in our study were highly specific. As can be seen in **S3 Fig**, methylation specific primers amplified methylated template only and vice versa. Negative control (water) showed no amplification with any of the primer pair. Altogether, MSP primers were found to be highly specific showing no cross reactivity **(S3 Fig)**.

**Fig 2:**
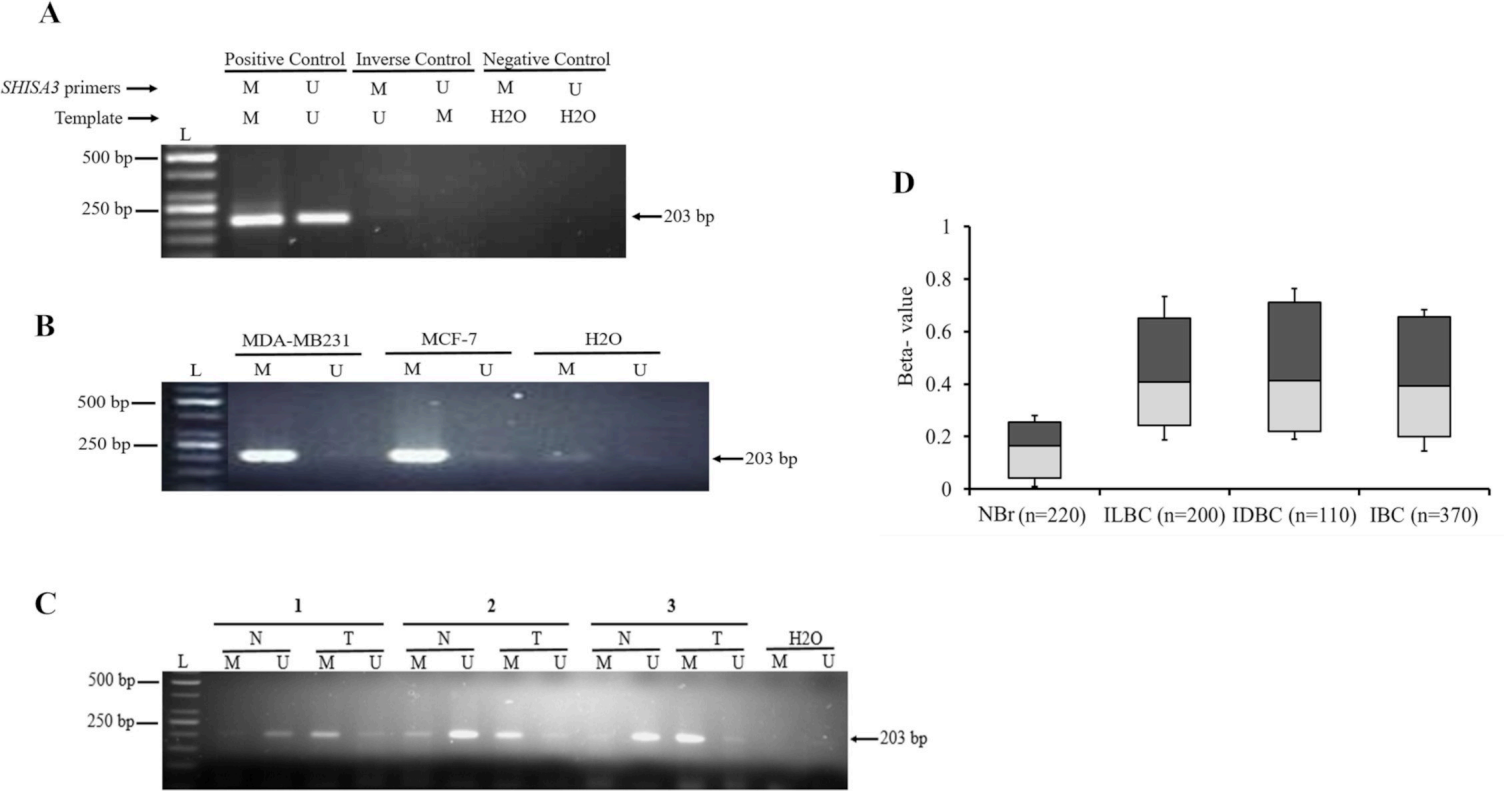
Methylation analysis of *SHISA3* promoter in breast cancer. A) Methylation analysis of *SHISA3* in breast cancer cell lines. B) Representative image of MSP of *SHISA3* gene in paired BC tissue samples. In all agarose gels images, L: DNA Ladder (50bp), N: normal tissue, T: tumor tissue, M: methylated, U: un-methylated, water was used as negative control for both methylated and un-methylated primers. C) *In silico* analysis of *SHISA3* methylation levels in normal and various breast cancer subtypes.

The MSP analysis showed *SHISA3* promoter hypermethylation in both MDA-MB231 and MCF-7 cell lines **(Fig 2A)**. MSP analysis of 103 paired BC tissues revealed that the frequency of the *SHISA3* promoter hypermethylation is significantly higher in BC tissues as compared to corresponding normal tissues. The *SHISA3* promoter was found methylated in 63 of 103 (61%) BC tissue samples. On the other hand, hypermethylation was observed only in 19 of 103 (18%) adjacent normal tissue samples. The agarose gel electrophoresis images of three representative tissue samples are shown in **Fig 2B.** Methylation array analysis of TCGA-BRCA datasets indicated hypermethylation of the *SHISA3* gene in breast cancer specimens ILBC (75%), IDBC (81%) and IBC (70%) as compared to normal breast tissue (**Fig 2C)**.

### 3.3 Restoration of *SHISA3* expression and methylation level after Aza treatment

**Fig 3:**
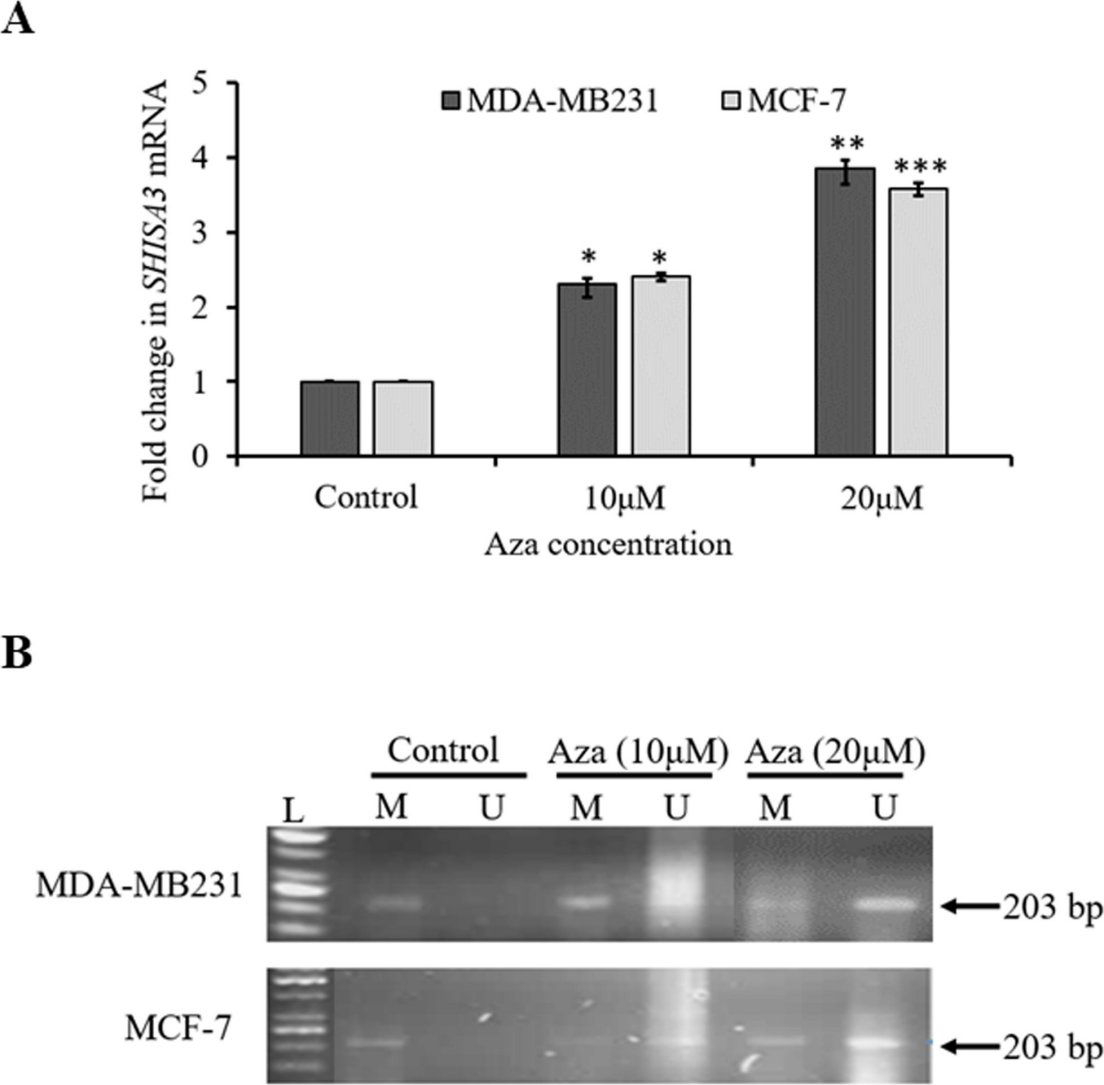
The mRNA expression and DNA methylation levels of *SHISA3* in BC cells before and after Aza treatment. (A) The qPCR analysis of *SHISA3* mRNA expression in BC cell lines before (control) and after treatment with 10 and 20μM Aza. (B) Methylation analysis of selected *SHISA*3 promoter region in MDA-MB231 (upper panel) and MCF-7 (lower panel) before and after treatment with Aza. L: DNA Ladder (50bp), M: methylated, U: un-methylated and Aza: 5-aza-2′-deoxycytidine.

In order to explore whether the downregulation of *SHISA3* was a consequence of its promoter hypermethylation in MDA-MB231 and MCF-7 cells were treated with 10 and 20μM of DNA methyl transferase inhibitor 5-aza-2′-deoxycytidine (Aza). Our results revealed that Aza treatment of both cell lines substantially enhanced *SHISA3* mRNA levels **(Fig 3A).** On the other hand, *SHISA3* promoter methylation level was significantly reduced in both BC cell lines after Aza treatment **(Fig 3B)**. Furthermore, change in expression and methylation in both cell lines were directly proportional to the dose of Aza. Taken together, our results indicated that promoter methylation is a major mechanism of *SHISA3* silencing in breast cancer cells.

### 3.4 Effect of *SHISA3* on cell proliferation and migration

**Fig 4:**
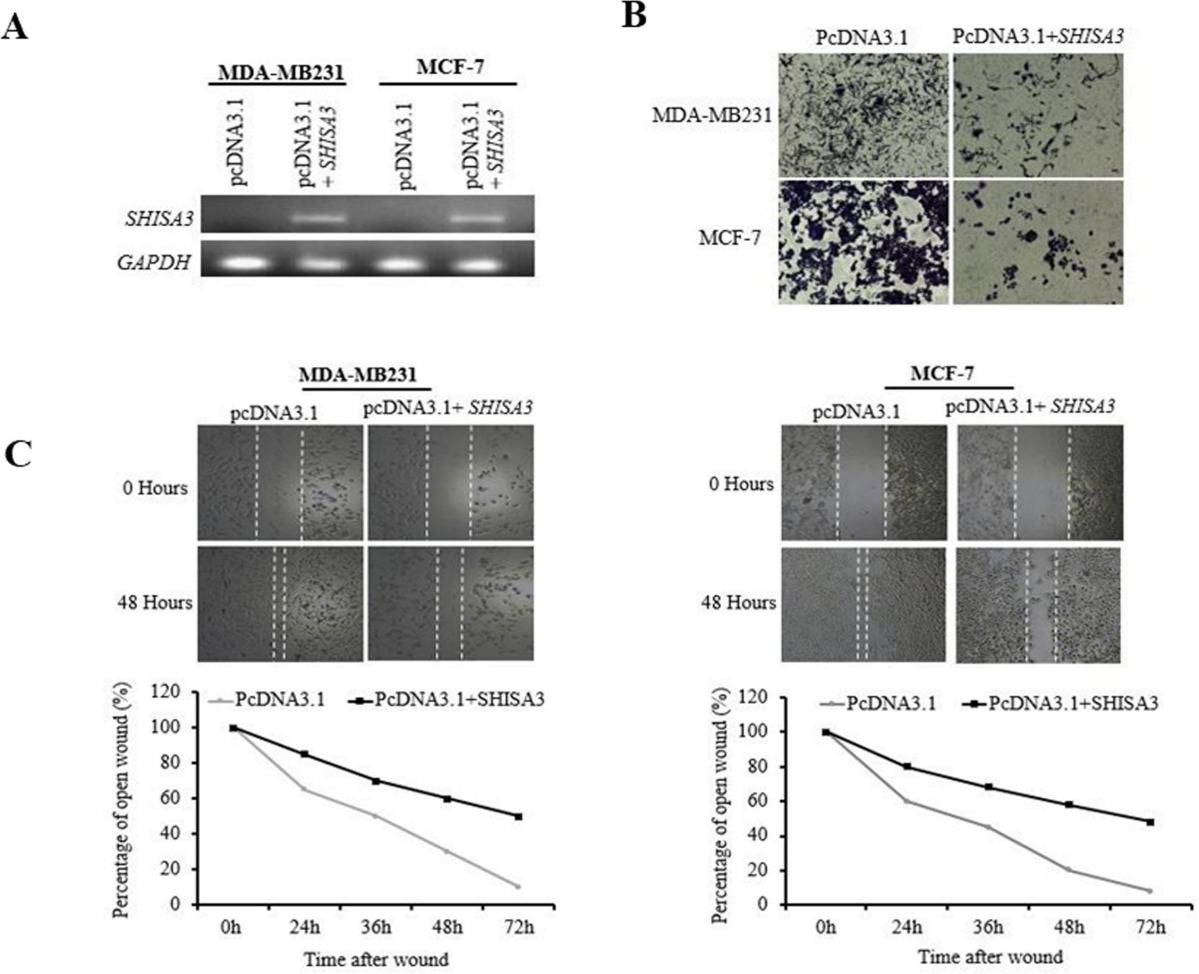
Breast cancer cells proliferation and migration abilities before and after *SHISA3* ectopic expression. A) qPCR of *SHISA3* expression in control (pcDNA3.1) and pcDNA3.1-*SHISA3* transfected MDA-MB231 and MCF-7 cells. GAPDH was used as internal control for normalized gene expression. B) Cell proliferation assay of MDA-MB231 and MCF-7 cell before and after transfection of *SHISA3*. C) Wound-healing assay for cell motility of empty vector- or *SHISA3*-expressing vector transfected MDA-MB231 and MCF-7 cells. Upper: Representative images of wound sealing at 0 h or 48 h after wound scratch. Lower: percentage of wound sealing compared with that of controls at each time point as indicated.

In order to further analyze the role of *SHISA3* in BC tumorigenesis, we performed cell proliferation and wound healing assays. The ORF of *SHISA3* was cloned and transiently expressed in both MDA-MB231 and MCF-7 cells **(Fig 4A)**. The crystal violet assay results indicated that ectopic expression of *SHISA3* significantly reduced the proliferation abilities of both cell lines (MCF-7 and MDA-MB231) **(Fig 4B)**. The wound healing assay results showed that ectopic expression of *SHISA3* markedly suppressed the migration ability of BC cells **(Fig 4C)**. The results of these experiments convincingly support the hypothesis that *SHISA3* act as a tumor suppressor gene in BC.

### 3.5 Association between *SHISA3* hypermethylation and clinicopathological parameters

We also investigated the connection between *SHISA3* methylation levels and clinicopathological parameters of BC patients such as age, tumor size, histological grade, tumor stage and lymph node metastasis. In our data, significant correlation was found only between *SHISA3* methylation and histological grade (P = 0.037) and lymph node metastasis (P = 0.016). However, we did not observe correlation of *SHISA3* methylation with any other clinicopathological features **(Table 2)**.

**Table 2:** Association between *SHISA3* hypermethylation and clinicopathological parameters of breast cancer patients.

Parameters	Number (n = 103)	*SHISA3* promoter methylation status		P value
		Methylated n (%)	Un-methylated n (%)	
Age (Years)				0.713
≤50	35	20 (57)	15 (43)	
>50	50	32 (64)	18 (36)	
Unknown	18	11 (61)	7 (39)	
Histological grade				**0.037**
Poorly	32	26 (75)	6 (25)	
Well /Moderate	71	37 (52)	34 (48)	
Lymph node metastasis				**0.016**
Yes	28	23 (82)	5 (18)	
No	63	34 (53)	29 (47)	
Unknown	12	9 (64)	5 (36)	
Clinical stage				0.664
I +II	44	27 (61)	17 (39)	
III+ IV	59	39 (66)	20 (34)	
Tumor size				0.579
≤5 cm	44	25 (58)	19 (42)	
≥5cm	38	28 (64)	10 (36)	
Unknown	21	13 (62)	8 (38)	

## 4. Discussion

Currently, mammography is considered the gold standard modality for breast cancer (BC) diagnosis. However, the balance between benefits achieved through early diagnosis and the possible risks of over diagnosis and over treatment is very narrow in case of mammography [23]. Although many advanced imaging modalities, for instance, magnetic resonance imaging (MRI) and positron emission tomography (PET) are now available, the diagnosis of BC particularly at early stage is still challenging. Identification of novel diagnostic biomarkers particularly those which can be used for early and highly specific detection as well as unambiguous prognosis are of extreme importance in cancer research nowadays. Altered DNA methylation patterns have proven to be an auspicious biomarkers and therapeutic targets with immense clinical potential [24, 25]. Numerous epigenetically silenced genes have been identified across various cancer types, [26] including BC [27, 28] and a handful of them are already on the path to clinical translation. Very recently, *SHISA3* was described as tumor suppressor gene in lung cancer [17]. Subsequently, promoter region of *SHISA3* was found to be hypermethylated in colorectal cancer [18], laryngeal squamous cells carcinoma [29] and nasopharyngeal carcinoma [19] suggesting the possibly ubiquitous role that *SHISA3* may play in carcinogenesis. Identification of such biomarkers that may be dysregulated in multiple cancers may help in developing broad spectrum diagnostic platforms and therapeutic targets. However, no study has been found that elucidates the biological role of *SHISA3* in BC. Therefore, main purpose of this study was to evaluate the role of *SHISA3* in BC. Our study for the first time described hypermethylation of *SHISA3* leading to its inactivation in the BC. We also found that the proliferation and migration abilities of BC cells were inhibited due to the ectopic expression of *SHISA3* which hints at its role as a tumor suppressor gene.

Previously, *SHISA3* has been described as an antagonist of *Wnt*/β-catenin pathway in lung cancer by reducing *Wnt* receptors, accelerating β-catenin degradation and ultimately suppressing *Wnt*-mediated gene expression [17]. Unusual activation of *Wnt* signaling pathway plays a vital role in breast cancer initiation and progression [30, 16]. Multiple studies have shown elevated β-catenin levels, suggesting its increased stability in a majority of BC tissues [31]. However, surprisingly N-terminal mutations of β-catenin often associated with its increased stabilization have not been observed in breast cancer. Similarly, inactivating mutations in *AXIN* and *APC* genes have also been found to be extremely rare in human breast cancer samples. These observations collectively suggest that in BC aberrant *Wnt* pathway activation may be a consequence of rise in the expression of positive (e.g. *Wnt1*, *Wnt10b*, *LRP6*, *FZD7*) or decrease in the expression of negative/antagonists (e.g. *Wnt5a*, *Wnt5bsFRP1*, *sFRP5*, *WIF1*) components of *Wnt* pathway [32, 15]. The qPCR results of current study demonstrated significant reduction in *SHISA3* expression among majority of BC tissues and cell lines compared to the normal mammary epithelial cells **(Fig 1)**. Furthermore, online transcriptome data substantiated our experimental findings where mRNA levels of *SHISA3* were found to be substantially lower in various subtypes (ILBC, IDBC and IBC) of BC as compared to the normal breast tissues. Based on these findings, it is tempting to speculate that *SHISA3* downregulation might be one of the possible mechanisms underlying anomalous activation of *Wnt* signaling in BC. However, a detail study may be needed to further explore the association between *SHISA3* silencing and *Wnt* activation in BC.

Altered DNA methylation patterns especially in promoter regions are known to regulate the expression of genes. Promoter hypermethylation is considered as responsible for the transcriptional inactivation of TSGs and has been proven as a crucial factor in initiation and progression of multiple cancer types [33, 6] including BC [27]. The *Wnt* pathway activation via epigenetic silencing of various *Wnt* antagonists has also been found in various cancers [34, 35, 36] including BC [37, 38, 39]. In this study, the *SHISA3* promoter was found hypermethylated in 61% (63/103) of BC tissue samples. On the other hand, hypermethylation was observed only in 18% (19/103) of adjacent normal tissue samples **(Fig 2)**. The *SHISA3* methylation frequency in our study was comparable to previous reports in colorectal cancer [18] and laryngeal squamous cell carcinoma [29]. Furthermore, methylation database analysis in this study revealed that the methylation level (β-values) of *SHISA3* gene are significantly higher in ILBC (75%), IDBC (81%) and IBC (70%) as compared to normal breast tissue. These data indicate that *SHISA3* promoter hypermethylation is a common event in BC and it is not restricted to a specific subtype suggesting *SHISA3* may be a potential diagnostic and prognostic biomarker for multiple cancer types. In order to find the correlation between promoter hypermethylation and silencing of *SHISA3* in BC, we treated BC cell lines with 5’-aza, 2-deoxycytidine (Aza), a well-known demethylating agent. *SHISA3* expression was retrieved in both BC cell lines, indicating that hypermethylation of *SHISA3* promoter was responsible for the silencing of this gene in BC, like many other types of cancer.

Cell motility, migration and invasion are the first and crucial steps involved in cancer metastasis [21]. Aberrant *Wnt* signaling has also been implicated in epithelial to mesenchymal transformation (EMT), and metastasis initiating cells. It has been shown that *Wnt* signaling induced EMT causes early (premalignant stage) dissemination of BC cells, indicating the strong correlation between *Wnt* activation and metastasis [40]. In line with this evidence, we sought to understand the possible role of *SHISA3* silencing in BC metastasis via *Wnt* pathway activation. We measured the proliferation and migration abilities of breast cancer cell lines (MCF-7 and MDA-MB231) after ectopic expression of *SHISA3*. Interestingly, re-expression of *SHISA3* significantly halted the proliferation and migration properties of both of these cell lines. However, the effect of *SHISA*3 was marginally variable in these cell lines which could be explained by the different molecular signature of these cell lines. As can be seen in Fig 4B and 4C, the effect of ectopic *SHISA3* expression on migration abilities of MDA-MB231 was more pronounced as compared to MCF-7. This could possibly be explained by the fact that MDA-MB231 represents a more aggressive TNBC type cell line. Previous studies have shown that β-catenin stabilization promotes cell migration, colony formation, and stemness in TNBC cells *in vitro* [41].

Finally, we tried to find out the link between *SHISA3* methylation and clinicopathological features of BC patients. It was observed that the *SHISA3* promoter methylation frequency was significantly higher in patients with poor differentiation (P = 0.037) and lymph node invasion (P = 0.016). However, no significant link was found with any other parameter studied.

Altogether, our study for the first time explored the epigenetic silencing of *SHISA3* rendering in breast cancer and has shown that *SHISA3* silencing via promoter hypermethylation is a frequent event in BC. The clinicopathological data revealed that *SHISA3* epigenetic inactivation is a crucial episode in breast cancer. This study provides a novel diagnostic and therapeutic target in BC diagnosis and treatment.

## 5. Conclusion

The current study is the first to describe the hypermethylation and silencing of *SHISA3* gene in breast cancer. The re-gain of *SHISA3* expression in breast cancer cells after 5’-aza, 2-deoxycytidine confirms that hypermethylation is responsible for the inactivation of this gene in breast cancer. Furthermore, our data revealed that the ectopic expression of *SHISA3* significantly reduces the ability of breast cancer cells to proliferate and migrate, beaconing its role as a tumor suppressor in breast cancer. Altogether, findings of this study may be useful in identifying a new target for the diagnosis and treatment of breast cancer.

## Supporting information

S1 Fig Histopathological characteristics of human breast (normal and cancerous) tissues. The tissue sections were stained with H & E and observed under microscope at 40X and 400X magnifications. Black, blue, brown and green arrows indicate tubular formation, musculo fibrous cells, tumor cells and nuclear pleomorphism respectively. Grade I tumor showed >75% tubular formation, mild nuclear pleomorphism and low mitotic count. Grade II tumor manifested 10–75% tubular formation, moderate nuclear pleomorphism and medium mitotic count. However, Grade III tumor revealed <10%, tubular formation, marked nuclear pleomorphism and high mitotic count.
(TIF)

S2 Fig Schematic flow chart indicating the pattern followed for online expression and methylation analysis of *SHISA3*. (TIF)

S3 Fig Analysis of *SHISA3* methylation primers specificity. The upper panel shows the primers used and the lower panel shows the template type.
(TIF)

S1 Raw images (PDF)
