# Supplementary figures and images for: SHISA3, an antagonist of the Wnt/β-catenin signaling, is epigenetically silenced and its ectopic expression suppresses growth in breast cancer
Source: PLoS One. 2020 Jul 21;15(7):e0236192. doi: 10.1371/journal.pone.0236192 (PMC7373276; doi:10.1371/journal.pone.0236192)

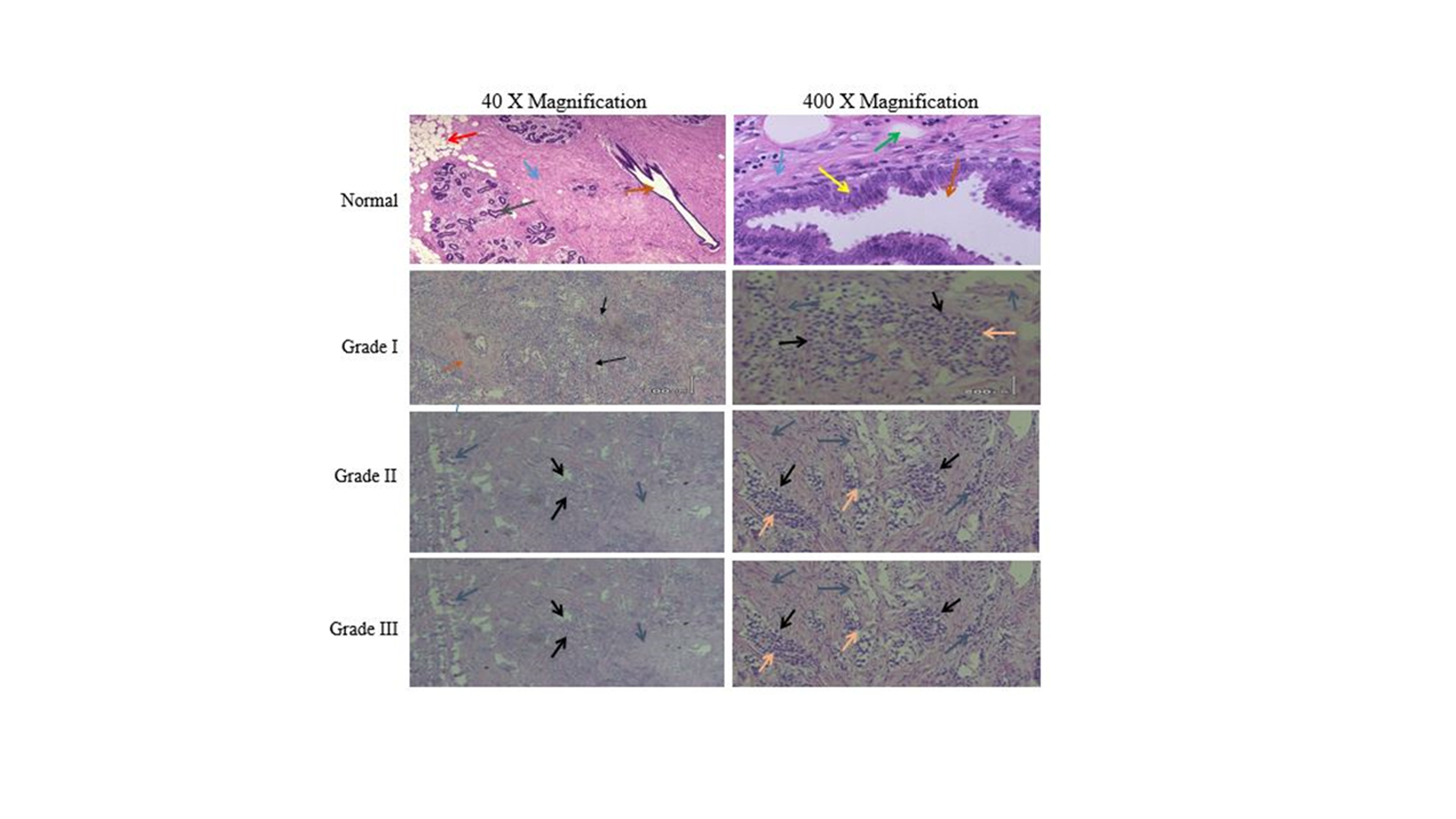

Supplement: S1 Fig — The tissue sections were stained with H & E and observed under microscope at 40X and 400X magnifications. Black, blue, brown and green arrows indicate tubular formation, musculo fibrous cells, tumor cells and nuclear pleomorphism respectively. Grade I tumor showed >75% tubular formation, mild nuclear pleomorphism and low mitotic count. Grade II tumor manifested 10–75% tubular formation, moderate nuclear pleomorphism and medium mitotic count. However, Grade III tumor revealed <10%, tubular formation, marked nuclear pleomorphism and high mitotic count. (TIF) [file pone.0236192.s001.tif]

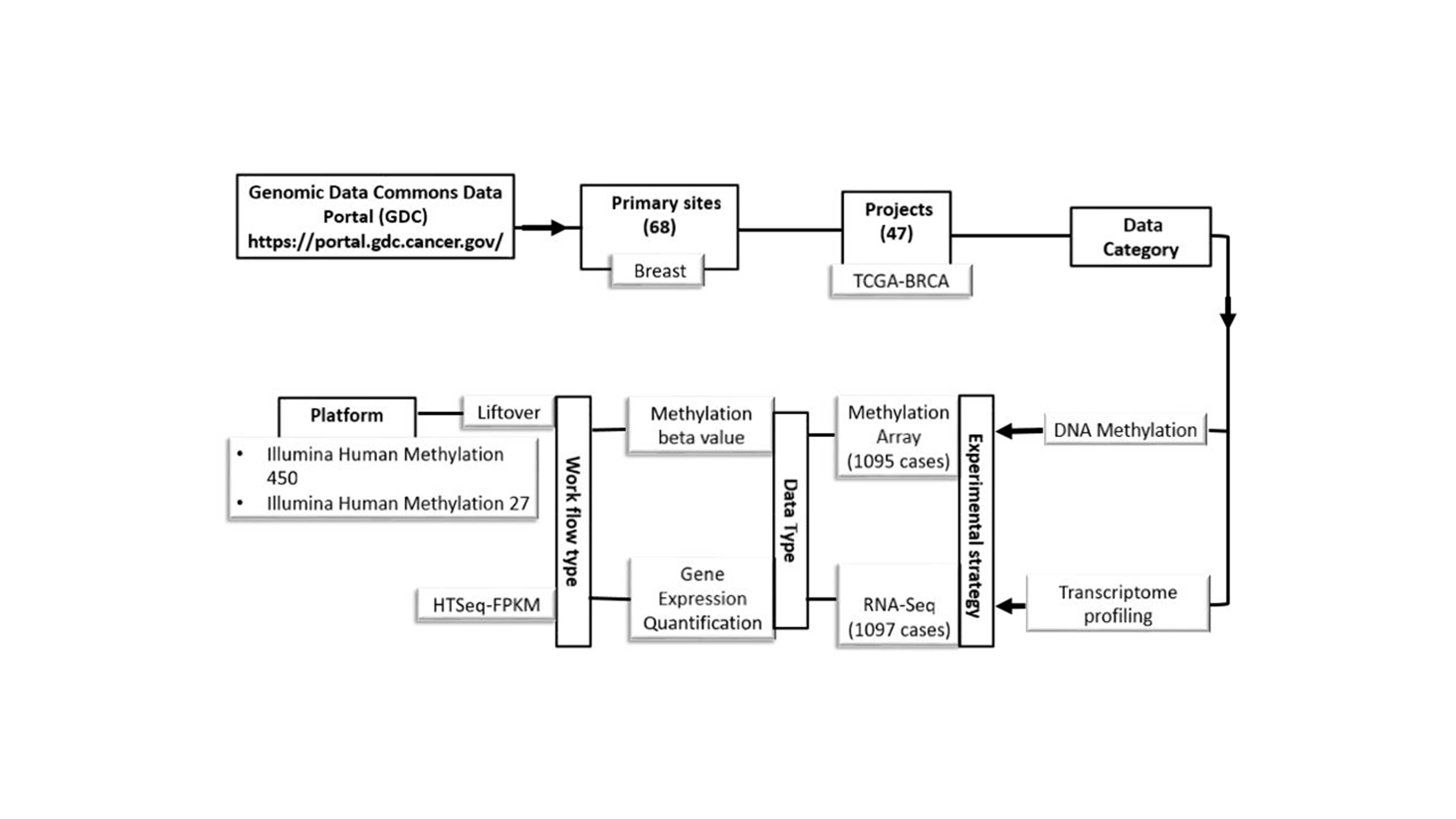

Supplement: S2 Fig — (TIF) [file pone.0236192.s002.tif]

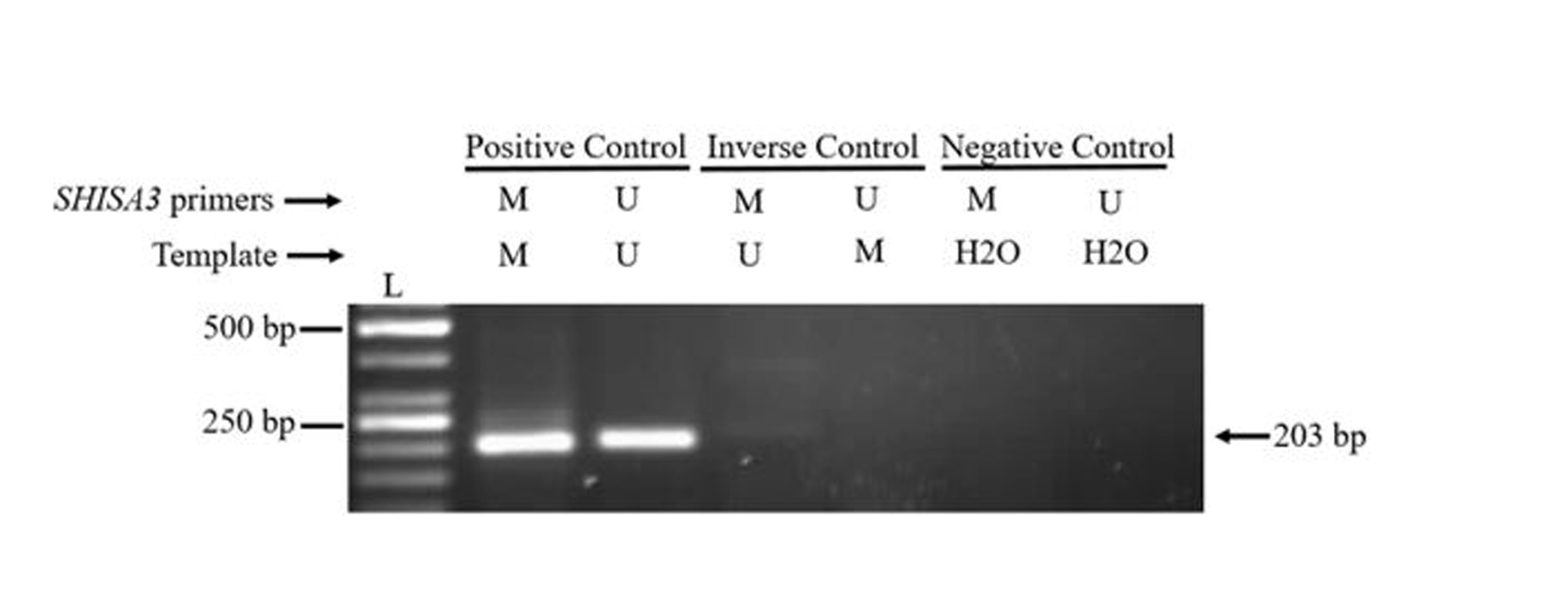

Supplement: S3 Fig — The upper panel shows the primers used and the lower panel shows the template type. (TIF) [file pone.0236192.s003.tif]

**Figure 2A**

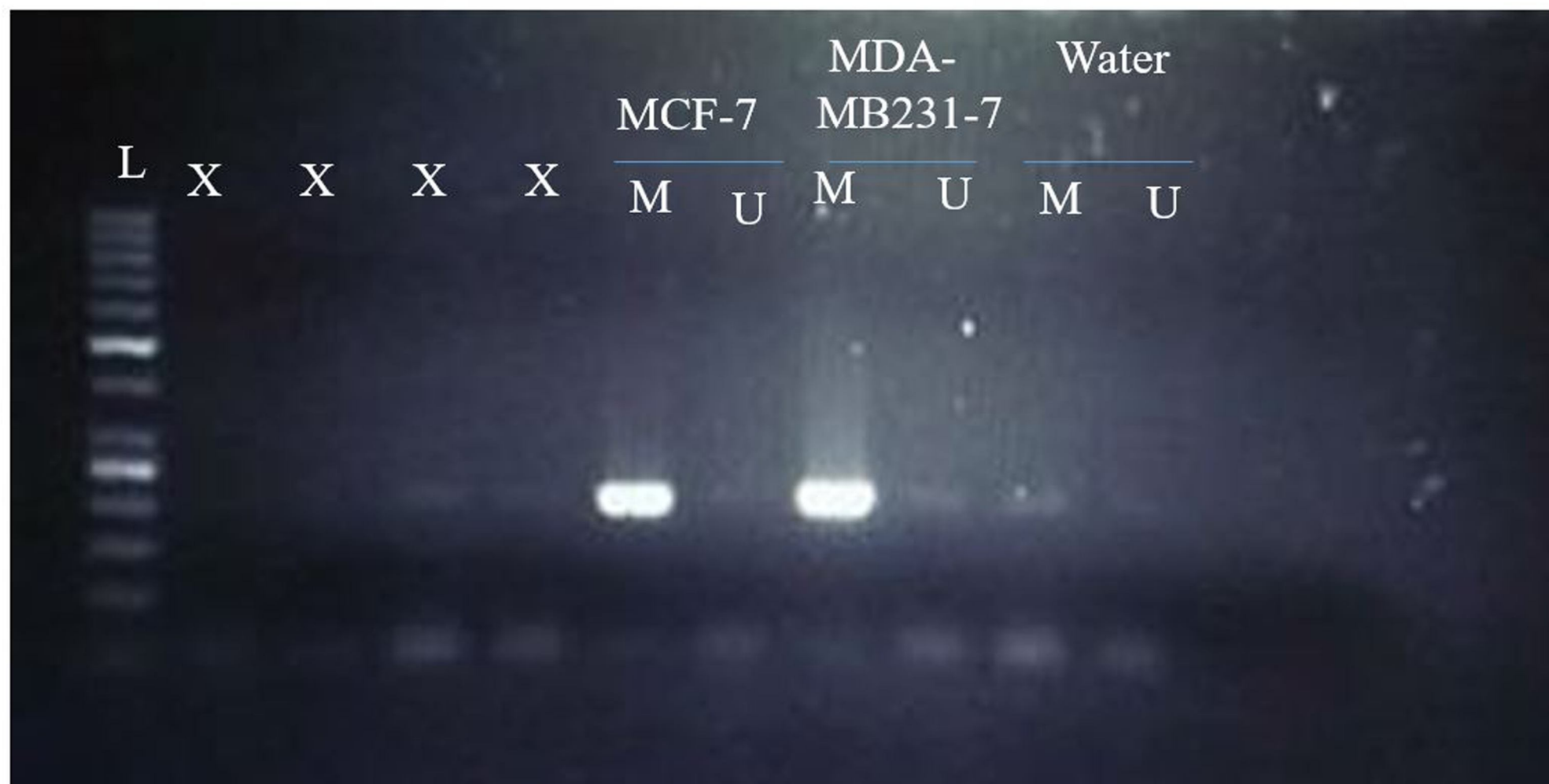

**Figure 2B**

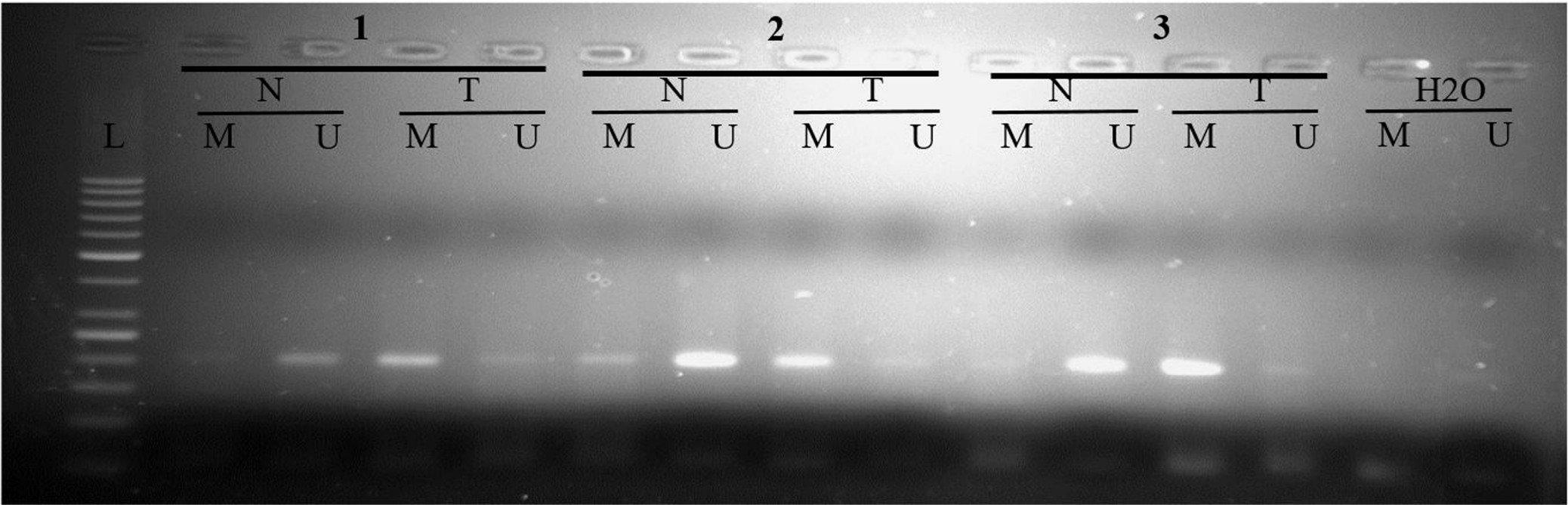

**Figure 3B**

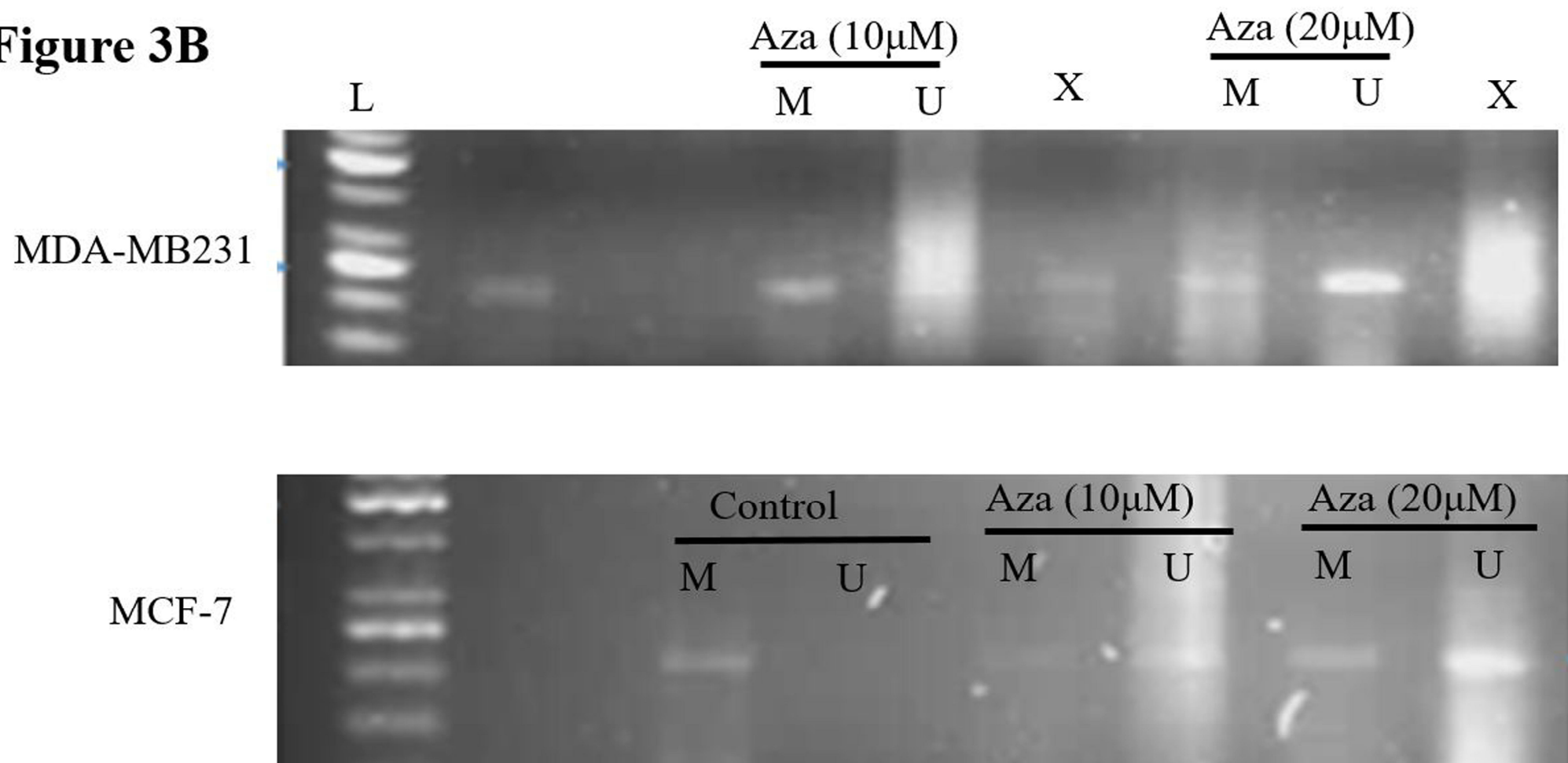

Figure 4A

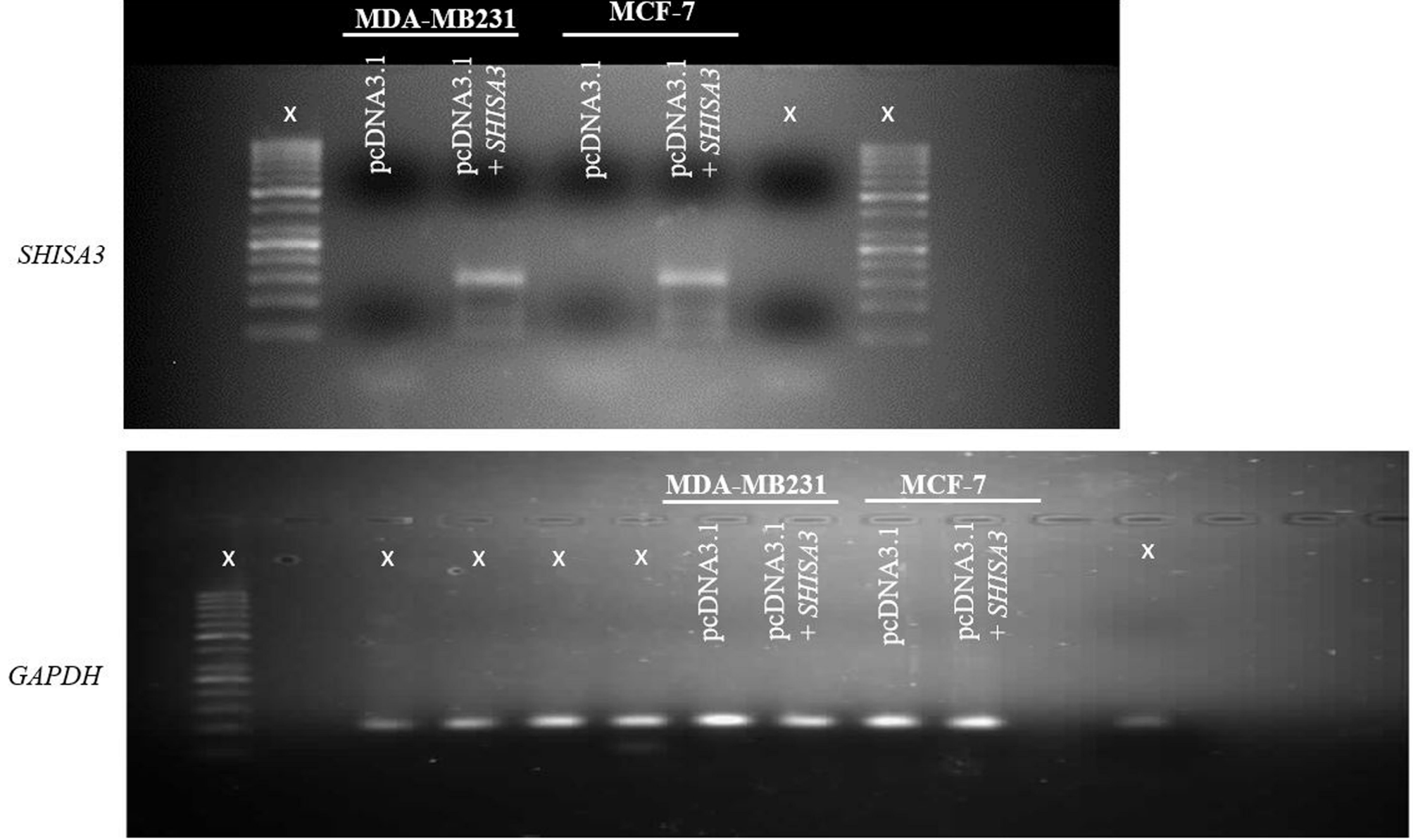

## Supplementary Figure 3

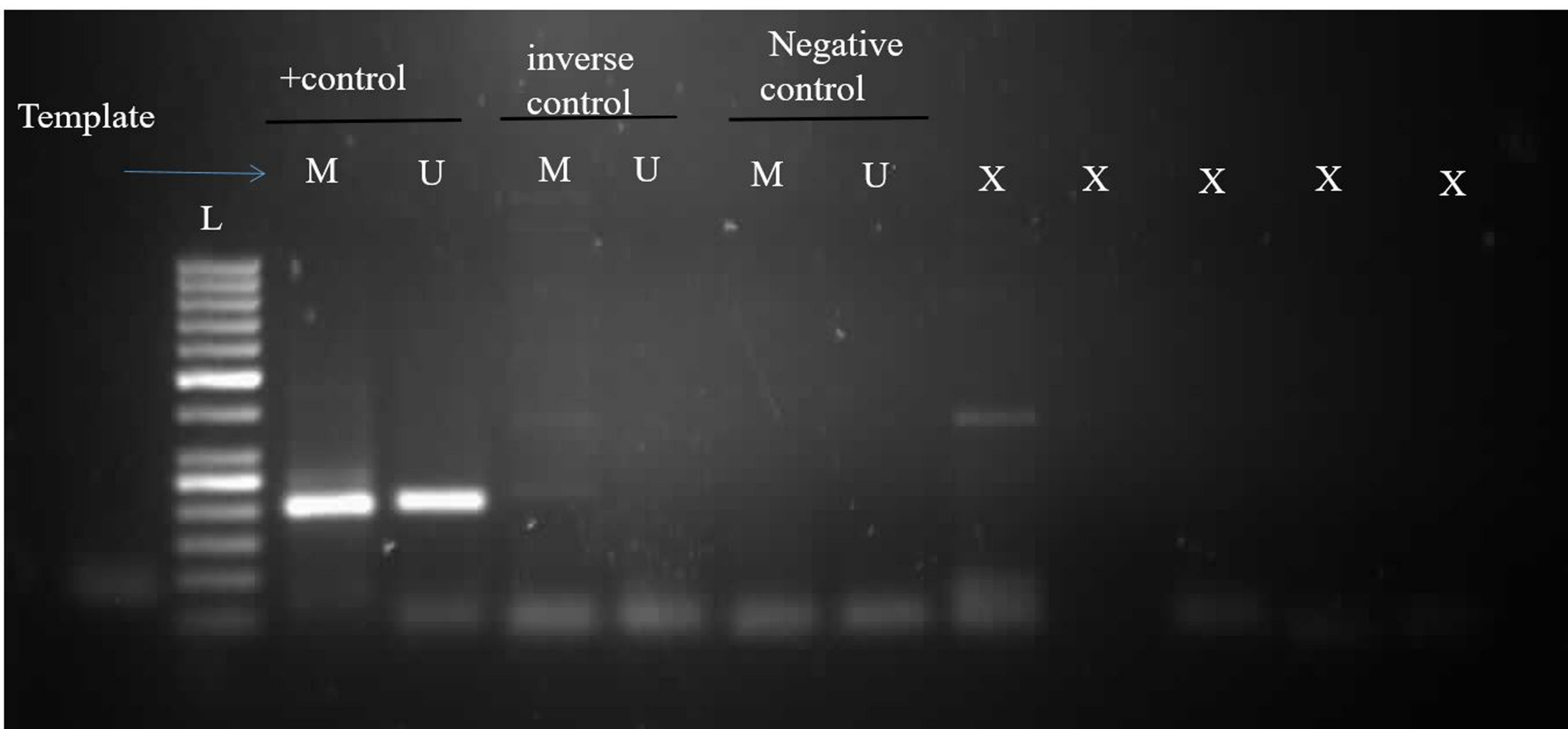

Supplement: S1 Raw images — (PDF) [file pone.0236192.s004.pdf]
